# Effect of total, domain-specific, and intensity-specific physical activity on all-cause and cardiovascular mortality among hypertensive adults in China

**DOI:** 10.1097/HJH.0000000000001601

**Published:** 2017-11-08

**Authors:** Mengyu Fan, Canqing Yu, Yu Guo, Zheng Bian, Xia Li, Ling Yang, Yiping Chen, Mingqiang Li, Xianzhi Li, Junshi Chen, Zhengming Chen, Jun Lv, Liming Li

**Affiliations:** a Department of Epidemiology and Biostatistics, School of Public Health, Peking University Health Science Center; b Chinese Academy of Medical Sciences, Beijing, China; c Clinical Trial Service Unit & Epidemiological Studies Unit (CTSU), Nuffield Department of Population Health, University of Oxford, Oxford, United Kingdom; d Liuzhou Center for Disease Control and Prevention; e Liuyang Center for Disease Control and Prevention, Liuyang; f China National Center for Food Safety Risk Assessment, Beijing, China

**Keywords:** Chinese, domain, hypertension, intensity, physical activity

## Abstract

**Objectives:**

We aimed to prospectively examine the associations of total, domain-specific, and intensity-specific physical activity with all-cause and cardiovascular mortality among Chinese hypertensive adults.

**Methods:**

We performed a prospective cohort study in 150 391 hypertensive participants aged 30–79 years from the China Kadoorie Biobank study of 512 891 participants recruited from 10 diverse areas across China during 2004–2008. Participants with heart disease, stroke, chronic obstructive pulmonary disease, and cancer at baseline were excluded.

**Results:**

During 1069 863 person-years of follow-up (median 7.1 years), a total of 5332 men and 4384 women died. Compared with hypertensive participants in the lowest level of total physical activity, the hazard ratios for all-cause mortality were 0.80 (0.76–0.84), 0.69 (0.65–0.73), and 0.67 (0.62–0.72) for those in quartiles 2–4 (*P*_trend_ *<* 0.001), respectively. Inverse associations were also observed for cardiovascular mortality. Being active in occupational, domestic, and leisure time were associated with lower risk of all-cause and cardiovascular mortality. However, the adjusted ratio for active commuting was 1.08 (1.02–1.15) for all-cause mortality. High levels of low-intensity, moderate-intensity, and vigorous-intensity physical activity were consistently associated with lower risks of all-cause and cardiovascular mortality.

**Conclusion:**

Among Chinese hypertensive adults, a higher level of physical activity reduces all-cause and cardiovascular mortality, independent of intensities of physical activity. Not only leisure-time but also occupational and domestic physical activities were benefited.

## INTRODUCTION

Hypertension is one of the most important risk factors for cardiovascular disease and premature mortality [1,2]. Nearly one in five Chinese adults suffers high blood pressure (BP), which equates to approximately 200 million hypertensive individuals [3]. Increasing mechanized and technologically driven lifestyles have led to rapidly reduced physical activity, even in less developed countries or areas. Increasing physical activity is a recommended lifestyle modification to manage hypertension, either in isolation or as an adjunct to antihypertensive medication. The benefits of regular physical activity influence multiple biological and emotional pathways that ultimately translates to health benefits across multiple diseases, notably a reduction in risk of cardiovascular disease mortality and all-cause mortality [4,5].

There are a variety of ways to expend energy, including occupational, commuting, domestic, and leisure-time activities. Although leisure-time physical activity is well documented to promote health [6], less is known about the impact of physical activity in other domains on health. Few studies have attempted to address these issues with inconsistent findings [7,8]. Given that the majority of employed people spend much of their day at work and that household chores are usually engaged in on a daily basis, the efforts to promote nonleisure time physical activity might be considered.

Current guidelines suggest at least 150 min of moderate-intensity activities or 75 min of vigorous-intensity activities throughout the week [9]. However, it is rather difficult to encourage sedentary groups, such as elder and hypertensive patients, to participate in vigorous exercise. A low-intensity physical activity program could increase adherence, but few studies have investigated the real impact of this physical activity intensity on cardiovascular performance. From a perspective of public health, it is crucial to quantify the health effects of physical activity from different intensities, particularly low-intensity activities.

Therefore, in the current study, we aimed to prospectively examine the associations of total, domain-specific, and intensity-specific physical activity with all-cause and cardiovascular mortality among Chinese hypertensive adults.

## METHODS

### Study design and participants

Data came from the China Kadoorie Biobank (CKB) study, a population-based prospective cohort of over 0.5 million Chinese adults. Details of the study design and sample characteristics have been described previously [10,11]. Briefly, a total of 512 891 men and women aged 30–79 years were recruited between 2004 and 2008 from 10 geographically defined areas (five urban and five rural) in China (Supplemental Material S1). The Ethical Review Committee of the Chinese Center for Disease Control and Prevention (Beijing, China) and the Oxford Tropical Research Ethics Committee, University of Oxford (UK) approved the study. Written informed consent was obtained from all participants.

For the current study, we included participants with prevalent hypertension at baseline. Prevalent hypertension was defined as measured SBP at least 140 mmHg, or measured DBP at least 90 mmHg, or self-reported physician-diagnosed hypertension, or self-reported use of antihypertensive medication at baseline. Of 180 612 hypertensive participants, we excluded those who reported a history of heart disease (*n* = 9574), stroke (*n* = 6814), chronic obstructive pulmonary disease (COPD; *n* = 15 398), or cancer (*n* = 973); those who reported both physical activity and sedentary leisure time equal to zero (*n* = 46); those who reported spending more than 20 h daily on all waking activities (including all physical activities and sitting activities, *n* = 211); and those who were lost to follow-up shortly after baseline (*n* = 2). The final analyses included 150 391 participants.

### Assessment of exposure

At baseline survey, the questions on physical activity and leisure sedentary time were adapted from validated questionnaires used in several other studies with some additional modifications after a CKB pilot study. As previously described [12,13], participants were asked about their usual type and duration for each of the four domains of physical activity (work, commuting, domestic, and leisure-time) and leisure hours per week spent sitting during the past year (Supplemental Material S2).

The updated 2011 Compendium of Physical Activities [14] was used to assign an intensity level of each activity (Supplemental Material S3). The level of each activity was calculated by multiplying its assigned metabolic equivalents (METs) value by hours spent on that activity per day. Domain-specific physical activity levels were calculated by summing all the MET-hours per day spent in occupational, commuting, domestic, and leisure-time, regardless of their intensity. Intensity-specific physical activity levels were calculated by summing all the MET-hours per day spent in low-intensity (<3.0 METs), moderate-intensity (3.0–5.9 METs), and vigorous-intensity (≥6.0 METs). The total physical activity level was calculated as a sum of all domain-specific or intensity-specific physical activity levels.

After completion of the baseline survey in July 2008, we randomly selected about 5% of the surviving participants in 10 survey sites for resurvey during August and October of 2008. To test the reproducibility of the total physical activity, we included 1300 participants who completed the same questionnaire twice at an interval of less than 1.5 years (median 1.4 years). The intraclass correlation coefficient (ICC) between the two questionnaires was 0.59.

### Assessment of covariates

Covariate information was collected in the baseline questionnaire including sociodemographic characteristics (age, sex, level of education, and marital status), lifestyle behaviors (alcohol consumption, smoking status, and intakes of red meat, fresh fruits, and vegetables), personal health and medical history (hypertension and diabetes), women's menopausal status, and information on family members, including biological parents and siblings who had had heart attack or stroke.

Baseline measurements of body weight, height, and BP were undertaken by trained staff using calibrated instruments. BP was measured at least twice on the unclothed right upper arm using a UA-779 digital monitor (A & D Instruments, Tokyo, Japan) after participants had remained at rest in a seated position for at least 5 min. If the SBP difference between the two measurements was more than 10 mmHg then a third measurement was made with the last two measurements were recorded. For the current analysis, we used mean of the two BP measurements. BMI was calculated as measured weight in kilograms divided by height in meters squared. Diabetes was defined either as a self-report of physician diagnosis of diabetes or screen-detected diabetes. Screen-detected diabetes was defined as no prior history of diabetes with a random blood glucose level at least 7.0 mmol/l and a fasting time more than 8 h; or a random blood glucose level at least 11.1 mmol/l and a fasting time less than 8 h; or a fasting blood glucose level at least 7.0 mmol/l.

### Ascertainment of death

The vital status was ascertained through local Disease Surveillance Points (DSP) system death registries, residential records, and active follow-up. Any deaths occurring among participants were coded using the 10th International Classification of Diseases by trained staff ‘blinded’ to baseline information. Primary study outcomes in the current study were all-cause mortality and cardiovascular mortality, with ischemic heart disease (IHD) (I20–I25) and cerebrovascular disease (I60–I69) as the underlying cause of death.

### Statistical analysis

Total physical activity level was categorized into four groups, based on quartiles among 150 391 hypertensive participants. Domain-specific and intensity-specific physical activity levels were dichotomized into higher and lower than the median physical activity. Baseline characteristics were adjusted for age and study region and compared across total physical activity categories using analysis of covariance for continuous variables and logistic regression for categorical variables. Person-years at risk were calculated from the recruitment date at baseline to the date of death, loss to follow-up, or 31 December 2013, whichever came first.

Hazard ratios and 95% confidence intervals (CIs) for all-cause and cardiovascular mortality associated with total, domain-specific, and intensity-specific physical activity levels were estimated using Cox proportional hazards regression models, with age as the underlying time metric. All multivariable analyses were stratified by age in 5-year intervals and by study region. Four multivariate models were fitted with different levels of adjustment for established and potential risk factors for mortality. Model 1 included age; Model 2 additionally included sex; level of education; marital status; alcohol consumption; smoking status; intake frequencies of red meat, fresh fruits, and vegetables; BMI; prevalent diabetes at baseline; family histories of stroke or heart attack (only in the corresponding analysis of cause-specific mortality); menopausal status (for women only). Model 3 additionally included SBP. Model 4 additionally included leisure sedentary time. For domain-specific and intensity-specific physical activity analyses, all the other domains or intensities were adjusted simultaneously in the model 4. Further, several sensitivity analyses were conducted to test the robustness of the results: excluding participants who died during the first 2 years of follow-up; excluding participants who had a BMI of less than 15 or more than 40; excluding participants who had diabetes at baseline; stratified the analyses by self-reported/screen-detected hypertension. We further excluded participants who died from transport accident for commuting physical activity analysis.

We also examined the associations of total physical activity with all-cause mortality among prespecified baseline subgroups based on age (<50, 50–59, and ≥60 years), smoking status (current regular smoker or not), alcohol consumption (current regular drinker or not), BMI (<24.0, 24.0–27.9, and ≥28.0 kg/m^2^), prevalent diabetes (yes or no), SBP (<140, 140–159, and ≥160 mmHg), work status (employed or unemployed), vigorous-intensity physical activity (0 or >0 MET-h/day), and leisure sedentary time (<3 or ≥3 h/day). Total physical activity was categorized into two groups at the median of 15.6 MET-h/day, which is equivalent to 312 min of moderate-intensity or 156 min of vigorous-intensity physical activity per day. The tests for interaction were performed using likelihood ratio tests by comparing models with and without cross-product terms between the baseline stratifying variable and dichotomized physical activity variable.

Analyses were conducted with Stata (version 13.0; StataCorp, College Station, Texas, USA). Statistical tests were two-sided, and *P* value less than 0.05 was considered to indicate statistical significance.

## RESULTS

### Total physical activity and lifestyle factors

Table 1 summarizes the baseline characteristics of 150 391 participants by level of total physical activity. Compared with participants in the lower quartiles of total physical activity, those in the higher quartiles were younger, more likely to be rural residents, had lower BMI and prevalence of diabetes, and spent less time sitting during leisure time. Occupational and moderate-intensity physical activity contributed to a major part of total physical activity, especially for those in the highest quartile of total physical activity.

### Total physical activity and mortality

We prospectively followed study participants for a median of 7.1 years. During 1069 863 person-years of follow-up, we documented 5332 deaths in men and 4384 deaths in women. In age-adjusted (model 1) and multivariable-adjusted analyses (model 2), the level of total physical activity was significantly associated with a reduced risk of all-cause mortality (Table 2). Further adjustments for SBP (model 3) and leisure sedentary time (model 4) did not substantially change the association of total physical activity with total mortality. Compared with hypertensive participants in the lowest level of physical activity group, the adjusted hazard ratios (95% CIs) for all-cause mortality were 0.80 (0.76–0.84), 0.69 (0.65–0.73), and 0.67 (0.62–0.72) for those in quartiles 2–4 (*P* *<* 0.001 for trend), respectively. We observed similar inverse associations of total physical activity with the risks of death due to IHD and cerebrovascular disease. Compared with participants in the lowest level of physical activity group, those in the highest group showed a 33% reduction in the risk of death from IHD and a 35% reduction in the risk of death from cerebrovascular disease. Total physical activity was consistently associated with decreased risks of all-cause and cardiovascular mortality in both men and women (all *P* for interaction with sex >0.05; Supplemental Table S4).

### Domain-specific physical activity and mortality

**FIGURE 1:**
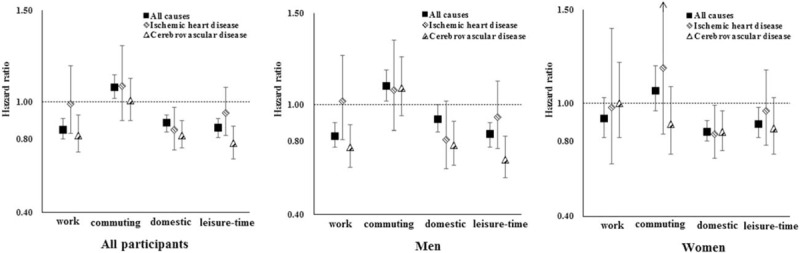
Associations between domain-specific physical activity and all-cause and cardiovascular mortality. Domain-specific physical activity was dichotomized by a median split, with low group used as a reference. Multivariate models were adjusted for: age; sex for all participants only; level of education; marital status; alcohol consumption; smoking status; intake frequencies of red meat, fruits, and vegetables; BMI; prevalent diabetes at baseline; family history of heart attack or stroke; menopausal status for women only; SBP; leisure sedentary time; and all the other domain-specific physical activity. Vertical lines represent 95% confidence intervals.

The associations between domain-specific physical activity and all-cause and cardiovascular mortality are presented in Fig. 1 and Supplemental Table S5. There was no heterogeneity between men and women except in the association of occupational physical activity with cerebrovascular mortality (*P* for interaction with sex = 0.023).

Participants who were active at work had significantly reduced risk of all-cause mortality in all participants and cerebrovascular mortality in men. Being active in the domestic domain was associated with lower risk of all-cause and cardiovascular mortality. Being active in leisure time was associated with lower risk of all-cause and cerebrovascular mortality. However, higher level of commuting activities was associated with an increased risk of all-cause mortality (hazard ratio = 1.08; 95% CI, 1.02–1.15). In addition, no statistically significant difference in the association between commuting physical activity and all-cause mortality was observed between urban and rural areas.

### Intensity-specific physical activity and mortality

**FIGURE 2:**
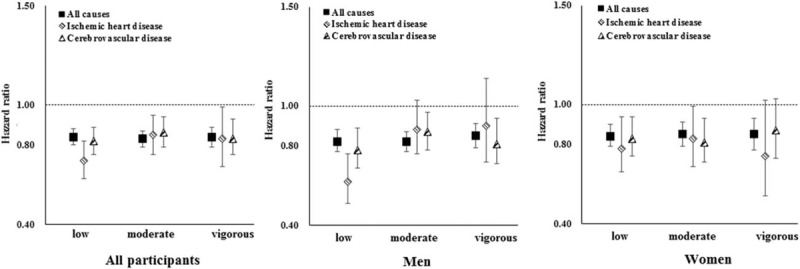
Associations between intensity-specific physical activity and all-cause and cardiovascular mortality. Intensity-specific physical activity was dichotomized by a median split, with low group used as a reference. Multivariate models were adjusted for: age; sex for all participants only; level of education; marital status; alcohol consumption; smoking status; intake frequencies of red meat, fruits, and vegetables; BMI; prevalent diabetes at baseline; family history of heart attack or stroke; menopausal status for women only; SBP; leisure sedentary time; and all the other intensity-specific physical activity. Vertical lines represent 95% confidence intervals.

High levels of low-intensity, moderate-intensity, and vigorous-intensity physical activity were consistently associated with lower risks of all-cause and cardiovascular mortality after adjusting for traditional risk factors and all other intensity-specific physical activity (Fig. 2 and Supplemental Table S6). Male participants showed a significantly stronger inverse association of low-intensity physical activity with IHD mortality (*P* for interaction with sex = 0.047). Statistically significant heterogeneity was also observed in the associations between vigorous-intensity physical activity and all-cause mortality (*P* for interaction = 0.029), and vigorous-intensity physical activity and cerebrovascular mortality (*P* for interaction = 0.016) by sex. Nevertheless, the associations seemed to be consistent between men and women.

### Sensitivity analyses

The associations of total, domain-specific, and intensity-specific physical activity with all-cause and vascular mortality were not changed substantially in sensitivity analyses with exclusion of participants dying during the first 2 years of follow-up; exclusion of participants who had a BMI of less than 15 or more than 40; and exclusion of participants with diabetes at baseline (data not shown). The associations between total physical activity and mortality were consistent between self-reported hypertension/self-reported medication and screen-detected hypertension. Furthermore, the association of commuting physical activity with all-cause mortality remained unchanged when we excluded participants who died from transport accident.

### Subgroup analyses

**FIGURE 3:**
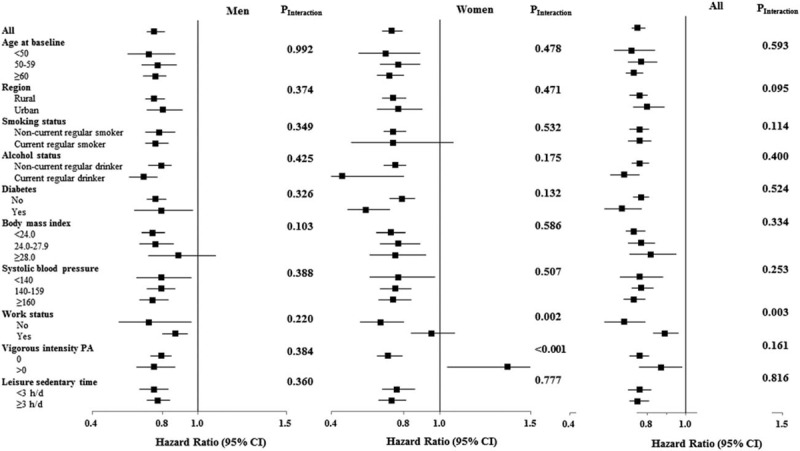
Subgroup analysis of associations between total physical activity and total mortality. Total physical activity was dichotomized by a median split (15.6 MET-h/day), with low group used as a reference 0251660288251659264. Multivariate models were adjusted for: age; sex for all participants only; level of education; marital status; alcohol consumption; smoking status; intake frequencies of red meat, fruits, and vegetables; BMI; prevalent diabetes at baseline; family history of heart attack or stroke; menopausal status for women only; SBP; and leisure sedentary time. Horizontal lines represent 95% confidence intervals. *P* values for interactions were computed with the use of likelihood ratio tests comparing Cox proportional-hazards models with and without cross-product terms for each level of baseline stratifying variables.

We also examined the associations between total physical activity and all-cause mortality according to baseline factors (Fig. 3 and Supplemental Table S7); the inverse associations were generally similar across subgroups stratified according to age, region, smoking status, alcohol consumption, BMI, prevalent diabetes, SBP, vigorous-intensity physical activity, and leisure sedentary time. Statistically significant differences across strata were observed for work status in all (*P* = 0.003 for interaction) and female participants (*P* = 0.002 for interaction), with a stronger inverse association in unemployed participants; and for vigorous-intensity physical activity in female participants (*P* < 0.001 for interaction), with increased risks of all-cause mortality were observed for high levels of total physical activity among those reporting any vigorous-intensity physical activity.

## DISCUSSION

In this large prospective study, among Chinese hypertensive middle-aged adults, we observed that being physically active was associated with significant reductions in risks of all-cause and cardiovascular mortality in both men and women. We observed similar magnitudes of risk reduction in all-cause and cardiovascular mortality associated with higher work-related, house-related, and leisure-time physical activity but increased risk of all-cause mortality associated with higher commuting-related physical activity. Intensity-specific physical activity levels were also associated with lower all-cause and cardiovascular mortality.

### Comparison with other studies and potential mechanism

Consistent with previous research conducted in Western population focusing on total physical activity or leisure-time physical activity [15], our findings showed a protective effect of total and leisure-time physical activity on all-cause and cardiovascular mortality. Possible mechanisms might involve the protective effect of activity on traditional cardiovascular risk factors [15,16], including the changes in BP, lipid levels, glucose tolerance, or BMI. Recent studies have indicated that physical activity has direct effects on both vasculature function and structure, known as ‘vascular deconditioning and conditioning’ effect, may therefore contribute to decreased cardiovascular risk [17]. Other potential benefits include improved endothelial cell function, attenuated plaque progression, stabilization of vulnerable plaques, reduction in myocardial oxygen demand, decreased thrombosis, and enhanced collateralization [18,19].

In addition to leisure-time physical activity, work-related and domestic physical activity is also important sources of energy expenditure. As observed in the present population, work-related physical activity contributed to a major part of total physical activity, especially for those in the highest quartile of total physical activity; and domestic physical activity accounted for a significant part of daily physical activity for physically inactive women. Only two studies on the possible role of work-related physical activity among hypertensive participants have yielded inconsistent results – protective [7] or no effect [8] on cardiovascular mortality. In the current study, we found beneficial effects of work-related physical activity on all-cause mortality and cerebrovascular mortality. We also showed that a high level of domestic physical activity was associated with significantly lower risks of all-cause and cardiovascular mortality among hypertensive participants. The cardiovascular benefits from domestic physical activity are in line with previous observation in general population [20]. Our results suggested that a broader range of physical activity that fits into everyday life could lead to substantial increase in the level of total physical activity and confer survival benefits in hypertensive individuals.

Recent studies have demonstrated that regular walking or cycling may reduce the risk of stroke [21], coronary heart disease [22], and mortality among general population [23,24]. Encouraging active commuting has become one of the most important strategies to increase physical activity level. In contrast to result from Hu *et al.*[7] showing beneficial effects of active commute on cardiovascular mortality among Finnish participants with hypertension, we found that high level of commuting physical activity was associated with an overall 8% increasing in the risk of all-cause mortality. The results did not change substantially with excluding participants who died from transport accident. Exposure to outdoor pollutants may be a possible explanation [25–27]. Further studies are needed, as the concern about air pollution is growing in China.

Current guidelines on physical activity highlight the benefits of both moderate and vigorous activities on health. However, emerging evidence indicates that low-intensity activities are as beneficial as moderate and vigorous physical activity [28]. In line with previous studies conducted in hypertensive men [29,30], our results showed that low-intensity, moderate-intensity, and vigorous-intensity physical activity were all related to the reduced risk of mortality due to all-cause and vascular specific diseases among both hypertensive men and women. Several previous studies have found that antioxidant capacity, resting heart rate, and small artery reactive hyperemia index improves with low-intensity physical activity [31]. Low-intensity physical activity should be encouraged to interrupt prolonged sitting, especially for the elderly and hypertension patients, considering it is easier to promote and adhere to. Some clinical trials have shown that exercise reduces BP in both hypertensive and normotensive adults [32,33]. In addition, physical activity is relatively less expensive intervention than the pharmacological treatment. Therefore, an increase in physical activity among hypertensive adults has important public health significance.

### Strengths and limitations of this study

The current study has several strengths. To our knowledge, this is the first large prospective study examining the associations of physical activity with all-cause and cardiovascular mortality in hypertensive adults. The physical activity was measured comprehensively across four domains and three intensities. We have carefully controlled for a wide range of established and potential risk factors for death. We also sought to minimize the potential bias arising from reverse causation by excluding participants with heart disease, stroke, cancer, or COPD who might abstain from exercise.

Some limitations of this study deserve attention. Physical activity was self-reported, potentially leading to measurement error because of recall bias and difficulties in capturing low-intensity activities. We only used baseline physical activity information and did not update it during follow-up. However, in a prospective study design, the observed associations are more likely to be underestimated because of the exposure misclassification and use of baseline measurements only [34]. In addition, we did not have data to characterize physical activity by type (isometric or isotonic). Despite the fact that we have controlled for several potentially confounding variables, residual confounding due to measurement error in confounders, use of baseline covariate measurements only, or other unmeasured factors was still possible.

In conclusion, in thus far the largest prospective cohort of the Chinese population, different domains of physical activity including leisure-time, occupational, and household were associated with substantial reductions in risks of all-cause and cardiovascular mortality among hypertensive adults. Low-intensity activity showed the similar protective effect on mortality as moderate-intensity and vigorous-intensity activities. Our findings provide new evidence that health benefits can be obtained through regular physical activity participation regardless of domain and intensity. These findings provide evidence that people can be encouraged to increase their activity levels for health benefit by any means necessary.

## ACKNOWLEDGEMENTS

The chief acknowledgment is to the participants, the project staff, and the China National Centre for Disease Control and Prevention (CDC) and its regional offices for assisting with the fieldwork. We thank Judith Mackay in Hong Kong; Yu Wang, Gonghuan Yang, Zhengfu Qiang, Lin Feng, Maigeng Zhou, Wenhua Zhao, and Yan Zhang in China CDC; Lingzhi Kong, Xiucheng Yu, and Kun Li in the Chinese Ministry of Health; and Sarah Clark, Martin Radley, Mike Hill, Hongchao Pan, and Jill Boreham in the CTSU, Oxford, for assisting with the design, planning, organization, and conduct of the study.

The study was supported by grants (81390544 and 81390541) from the National Natural Science Foundation of China. The CKB baseline survey and the first resurvey were supported by a grant from the Kadoorie Charitable Foundation in Hong Kong. The long-term follow-up is supported by grants from the UK Wellcome Trust (088158/Z/09/Z and 104085/Z/14/Z); by a grant from the Chinese Ministry of Science and Technology (2011BAI09B01). The funders had no role in the study design, data collection, data analysis and interpretation, writing of the report, or the decision to submit the article for publication.

### Conflicts of interest

There are no conflicts of interest.

## Supplementary Material

Supplemental Digital Content

## Figures and Tables

**TABLE 1:** Baseline characteristics of 150 391 hypertensive participants by sex and level of total physical activity^a^

	Men, *n* = 64 287)				Women, *n* = 86 104			
	Q1	Q2	Q3	Q4	Q1	Q2	Q3	Q4
No. of participants	16 872	11 822	15 966	19 627	20 756	25 751	21 628	17 969
Age (year)	61.3	56.3	53.0	50.3	60.3	57.5	53.3	49.9
Urban (%)	55.8	50.3	38.5	36.2	50.6	45.8	35.9	27.1
Middle school and above (%)	55.7	57.0	55.0	49.8	29.7	31.2	31.3	29.3
Married (%)	92.5	91.0	92.1	92.6	83.9	86.6	86.9	87.2
Current regular smoker (%)	56.3	57.7	58.4	59.7	2.3	1.9	2.1	1.7
Current regular drinker (%)	33.7	37.9	38.9	38.6	1.6	1.7	2.1	2.1
Average intake frequency (day/week)^b^								
Red meat	3.9	3.9	3.9	3.9	3.2	3.2	3.1	3.3
Vegetable	6.9	6.9	6.8	6.9	6.8	6.9	6.8	6.8
Fruit	2.2	2.2	2.0	1.9	2.2	2.4	2.3	2.2
Postmenopause (%)	–	–	–	–	71.4	70.7	70.2	70.2
BMI (kg/m^2^)	24.8	24.6	24.4	24.1	25.2	25.1	24.7	24.6
SBP (mmHg)	151.3	151.0	151.9	152.2	153.2	152.9	153.0	153.3
Prevalent diabetes (%)	9.8	8.6	7.4	6.2	12.7	10.8	8.7	6.6
Family history of (%)								
Stroke	24.6	25.3	25.2	25.0	23.7	25.4	24.7	24.0
Heart attack	7.7	7.6	7.7	7.4	6.8	7.0	7.0	6.3
Leisure sedentary time (h/day)	3.6	3.2	2.9	2.6	3.2	3.1	2.6	2.4
Levels of PA (MET-h/day)								
Total PA	5.2	12.8	21.2	39.9	7.4	12.5	20.9	38.4
Occupational PA^c^	3.0	9.1	15.9	33.8	4.3	5.6	11.4	27.1
Commuting PA^c^	1.0	1.6	2.1	2.9	1.1	1.4	1.9	2.8
Domestic PA	2.1	3.4	3.3	3.1	5.9	8.8	9.2	8.2
Leisure-time PA	0.6	1.4	1.2	1.0	0.1	1.3	1.3	0.9
Low PA	3.2	8.6	10.5	4.3	6.3	9.8	14.4	10.9
Moderate PA	1.4	2.9	7.9	28.6	0.6	1.9	3.8	19.7
Vigorous PA	0.6	1.2	2.8	7.0	0.5	0.8	2.6	7.8

MET, metabolic equivalent; PA, physical activity.

^a^Level of total PA was divided into four groups by quartiles, with Q1 as the lowest quartile group. Baseline characteristics were presented as mean or percentage, adjusted for age and study region, as appropriate. Tests for linear trend across PA categories were performed by using continuous PA variable in a separate regression model. All *P* for trend were <0.05, except for married in men (*P* = 0.278), stroke family history (men: *P* = 0.640; women: *P* = 0.155), heart attack family history in men (*P* = 0.058), and leisure-time PA in women (*P* = 0.137).

^b^Average intake frequencies of red meat, fresh vegetables, and fruits were calculated by assigning participants to the midpoint of their consumption category.

^c^Among working participants only.

**TABLE 2:** Associations between total physical activity and all-cause and cardiovascular mortality among 150 391 hypertensive participants

	Level of total physical activity^a^				
Causes of death	Q1	Q2	Q3	Q4	*P* for trend^b^
No. of person years	260 522	267 156	270 931	271 255	
All causes					
No. of deaths	3993	2369	1913	1431	
Model 1	1.00	0.74 (0.70–0.78)	0.70 (0.66–0.75)	0.72 (0.67–0.77)	<0.001
Model 2	1.00	0.80 (0.76–0.84)	0.69 (0.65–0.74)	0.68 (0.63–0.73)	<0.001
Model 3	1.00	0.80 (0.76–0.85)	0.69 (0.65–0.74)	0.67 (0.63–0.72)	<0.001
Model 4	1.00	0.80 (0.76–0.84)	0.69 (0.65–0.73)	0.67 (0.62–0.72)	<0.001
Ischemic heart disease					
No. of deaths	694	369	222	149	
Model 1	1.00	0.72 (0.63–0.82)	0.68 (0.58–0.81)	0.70 (0.57–0.86)	<0.001
Model 2	1.00	0.78 (0.69–0.89)	0.69 (0.59–0.82)	0.70 (0.57–0.86)	<0.001
Model 3	1.00	0.78 (0.69–0.89)	0.69 (0.59–0.82)	0.69 (0.57–0.85)	<0.001
Model 4	1.00	0.78 (0.68–0.88)	0.68 (0.57–0.80)	0.67 (0.55–0.83)	<0.001
Cerebrovascular disease					
No. of deaths	1152	656	545	405	
Model 1	1.00	0.71 (0.64–0.78)	0.66 (0.59–0.73)	0.67 (0.59–0.77)	<0.001
Model 2	1.00	0.76 (0.69–0.84)	0.64 (0.58–0.72)	0.65 (0.57–0.74)	<0.001
Model 3	1.00	0.76 (0.69–0.84)	0.64 (0.58–0.72)	0.65 (0.57–0.74)	<0.001
Model 4	1.00	0.76 (0.69–0.84)	0.64 (0.58–0.72)	0.65 (0.57–0.74)	<0.001

Multivariate models were adjusted for: model 1: age (years); model 2: additionally included sex; level of education (no formal school, primary school, middle school, high school, college, or university or higher); marital status (married, widowed, divorced or separated, or never married); alcohol consumption (nondrinker, occasional drinker, former drinker, or regular drinker); smoking status (never smoker, occasional smoker, former smoker, or regular smoker); intake frequencies of red meat, fruits, and vegetables (daily, 4–6, 1–3 days/week, monthly, or rarely or never); BMI; prevalent diabetes at baseline (presence or absence); family history of heart attack or stroke (presence or absence, only adjusted for in corresponding analysis of cause specific mortality); and menopausal status for women only; model 3: additionally included SBP (mmHg); model 4: additionally included leisure sedentary time (h/day).

^a^Level of total physical activity was divided into four groups by quartiles, with Q1 as the lowest quartile group.

^b^Tests for linear trend across physical activity categories were performed by using the continuous physical activity variable in a separate regression model.

## Reviewers’ Summary Evaluations

### Reviewer 1

Physical exercise is beneficial in unselected populations. Little is known about the effect of physical exercise in hypertensive subjects. In the present study, physical exercise was determined by questionnaire in 150,000 hypertensive subjects followed for 7 years. Morbidity and mortality during follow-up was determined from register data. The authors found a beneficial effect of any level of physical exercise on total and cardiovascular mortality and morbidity. Thus, it can be noted that physical exertion has similar effect in hypertensives as in the general population.

### Reviewer 2

The aim of the study was to assess how different types and grades of physical activity relate to mortality in Chinese population with hypertension. The authors observed that the lowest level of physical activity was associated with increased mortality. The strengths of the study are the large population and different parameters that were assessed. However, the novelty is limited as it is well known that physical activity reduces mortality. Moreover, the authors included only hypertensive patients and did not test the hypothesis whether the protective effects of physical activity are stronger among hypertensive individuals.
