# Supplementary material for: Effect of total, domain-specific, and intensity-specific physical activity on all-cause and cardiovascular mortality among hypertensive adults in China
Source: J Hypertens. 2017 Nov 8;36(4):793–800. doi: 10.1097/HJH.0000000000001601 (PMC5862003; doi:10.1097/HJH.0000000000001601)
Supplement: Supplemental Digital Content [file jhype-36-793-s001.doc]

# Supplemental Material S1

China Kadoorie Biobank baseline study took place during 2004–2008 in 10 geographically defined regions (5 urban and 5 rural) of China. The areas were selected according to local disease patterns, spectra of risk exposures, population stability, quality of death and disease registries, local commitment and capacity, but were not intended to be representative the general population (although within each study area the participants were relatively unselected).

In the 10 study areas, all potentially eligible participants aged 35–74 who are permanently resident (for complete follow-up of mortality and morbidity) and without major disability in each administrative unit (village or street committee) were identified through public registry records. Invitation letters and study information leaflets were delivered door-to-door by local community leaders or health workers, following extensive publicity campaigns. To encourage participation, those few individuals aged 30–34 or 75–79 years were not turned away. About one in three of the eligible residents (33% rural, 27% urban) participated.

# Supplemental Material S2. China Kadoorie Biobank study questionnaire on physical activity and sedentary leisure time*

## Section A: For non-farmers

1. In the past 12 months, how active were you at work?

🞎 Mainly sedentary (e.g. office worker)

🞎 Standing occupation (e.g. guard, shop assistant)

🞎 Manual work (e.g. plumber, carpenter)

🞎 Heavy manual work (e.g. miner, construction worker)

🞎 Retired, housewife/husband, unemployed, or disabled 🡪 *go to Question 12*

2. In a typical week, about how many hours did you usually work? hrs

3. In the past 12 months, how did you usually get to and from work?

🞎 Mainly walk ed 🞎 By bicycle

🞎 By motorbike 🞎 By bus/car/ferry/train

🞎 Mainly stayed at home, or work near home 🡪 *go to Question 12*

4. How much time did you spend each day on the journey to and from work?

mins

## Section B: For farmers

5. In the past 12 months, did your farming work change seasonally?

🞎 No 🡪  *go to Question 7*

🞎 Yes

6. In the farming season in the last 12 months:

- How many months did the farming season usually last? mths
- What types of farming work did it usually involve?

🞎 Manual 🞎 Semi-mechanized 🞎 Fully mechanized

- How many hours did you usually work each day? hrs
- Of which, how many hours did you sweat or have a much faster heartbeat?

hrs

7. In a typical week (in non-farming seasons), how many hours did you usually work in the field? hrs

8. Apart from the agriculture work, did you have any other job?

🞎 No 🡪 *go to Question 11*

🞎 Yes

9. How active were you at work with the other job?

🞎 Mainly sedentary 🞎 Mainly general manual work

🞎 Mainly standing 🞎 Mainly heavy manual work

10. In a typical week, about how many hours did you spent at the other job? hrs

11. In a typical day how much time did you usually spend on the journey to and from work on foot or by bicycle? mins

## Section C: For both farmers and non-farmers

12. In the past 12 months, how often did you exercise in your leisure time?

🞎 Never or almost never

🡪 *go to Question 15*

🞎 1-3 times/month

🞎 1-2 times/week 🞎 3-5 times/week

🞎 Daily or almost every day

13. What is your main type of exercise? *(tick one box only)*

🞎 Taichi /Qigong /Leisure walking 🞎 Brisk walking /Gymnastics /Folk dancing

🞎 Jogging /Aerobic exercise 🞎 Swimming

🞎 ‘Ball’ games (including also billiards, bowling, golf, tennis, table tennis and badminton)

🞎 Other (eg. mountain climbing, home exercises, and rope jumping)

14. About how many hours per week did you spent on these exercises? ___ hrs

15. In the past 12 months, how often did you sweat or have a much faster heartbeat because of exercise?

🞎 Never or almost never

🡪 *go to Question 17*

🞎 <1 time / week

🞎 1-2 times/week 🞎 3-5 times/week

🞎 Daily or almost every day

16. About how many hours per week did you do such vigorous activities? ____hrs

17. In the past 12 months, about how many hours per week did you do housework? ____ hrs

18. In the past 12 months, during leisure time, about how many hours per week did you spend on sitting activities (e.g. watching TV or reading)? hrs

19. How many hours do you typically sleep per 24h day (incl. naps)? hrs

* Information can also be found at <http://www.ckbiobank.org/about-the-study/study-design>

# Supplemental Table S3. Physical activity types, MET values, codes and intensity categories*

| **Activity type** | **Intensity** | **MET** | **Codes*** |
| --- | --- | --- | --- |
| Heavy manual work | Vigorous | 6.5 | 11477 |
| Manual work | Moderate | 4.5 | 11476 |
| Standing work | Moderate | 3.8 | Mean of 11610 and 11630 |
| Sedentary work | Low | 1.8 | Mean of 11580, 11585, and 11590 |
| Manual work in the farming season | Vigorous | 6.3 | Mean of 11145 and 11146 |
| Semi-mechanized work in the farming season | Moderate | 3.4 | Mean of 11146 and 11147 |
| Fully mechanized work in the farming season | Low | 2.4 | Mean of 11147 and 11170 |
| Work outside the farming season | Low | 2 | 11147 |
| Walking | Moderate | 4 | 17270 |
| Bicycle | Vigorous | 6.8 | 1011 |
| Motorbike | Moderate | 3.5 | 16030 |
| Private or public transportation (such as bus, car, underground, and ferry) | Low | 1.7 | Mean of 16010, 16015, and 16016 |
| Household activity | Low | 2.8 | Mean of 05030^†^, 05040^†^, 05035, 05055, 05070, 05090^†^, 05092^†^, 05184, 05197, and 05200 |
| Tai-Chi/qigong/leisure walking | Moderate | 3.3 | Mean of 15670 and 17160 |
| Jogging/aerobic exercise | Vigorous | 7.4 | Mean of 03015, 12020, and 12150 |
| Ball games | Moderate | 5.5 | Mean of 15020^†^, 15030^†^, 15055, 15080, 15090, 15255, 15605^†^, 15610^†^, 15652, 15660, 15675, 15710^†^, and 15711^†^ |
| Brisk walking/gymnastics/folk dancing | Moderate | 4.2 | Mean of 03025, 15300, and 17200 |
| Swimming | Vigorous | 7.2 | Mean of 18230, 18240, and 18310 |
| Other exercise, e.g. mountain walking, home exercise and rope jumping | Moderate | 5.9 | Mean of 02010, 02064, 04001, 04100, 15110^†^, 15120^†^, 15200, 15240, 15310, 15425^†^, 15430^†^, 15537, 15550^‡^, 15551^‡^, 15552^‡^, 15580, 15590, 15730, 15732^‡^, 15733^‡^, 15734^‡^, and 19030 |
| MET: Metabolic equivalent of tasks. * Based on the 2011 Compendium of Physical Activities: a second update of codes and MET values. Ainsworth BE, et al. Medicine and Science in Sports and Exercise, 2011;43(8):1575-1581. | | | |
| ^†^ Assigned 1/2 weight in calculating the mean MET value because the connecting two items represent one type of activity. | | | |
| ^‡^ Assigned 1/3 weight in calculating the mean MET value because the connecting three items represent one type of activity. | | | |

# Supplemental Table S4. Associations between total physical activity and all-cause and cardiovascular mortality by sex*

| Causes of death | |  | **Men** | | | |  |  | **Women** | | | | |
| --- | --- | --- | --- | --- | --- | --- | --- | --- | --- | --- | --- | --- | --- |
|  |  |  | Q1 | Q2 | Q3 | Q4 | P for trend† |  | Q1 | Q2 | Q3 | Q4 | P for trend† |
| No. of person years | |  | 114 596 | 83 161 | 112 909 | 140 533 |  |  | 145 926 | 183 994 | 158 022 | 130 722 |  |
| **All causes** | |  |  |  |  |  |  |  |  |  |  |  |  |
|  | No. of Deaths | | 2227 | 1037 | 1136 | 922 |  |  | 1766 | 1332 | 777 | 509 |  |
|  | Model 1 |  | 1.00 | 0.81 (0.75-0.88) | 0.74 (0.68-0.80) | 0.69 (0.63-0.76) | <0.001 |  |  | 0.75 (0.70-0.81) | 0.63 (0.58-0.69) | 0.61 (0.54-0.68) | <0.001 |
|  | Model 2 |  | 1.00 | 0.81 (0.75-0.88) | 0.73 (0.67-0.79) | 0.68 (0.62-0.74) | <0.001 |  |  | 0.79 (0.73-0.85) | 0.66 (0.60-0.73) | 0.66 (0.58-0.74) | <0.001 |
|  | Model 3 |  | 1.00 | 0.82 (0.76-0.88) | 0.73 (0.67-0.79) | 0.67 (0.62-0.74) | <0.001 |  |  | 0.79 (0.73-0.85) | 0.67 (0.61-0.73) | 0.66 (0.58-0.74) | <0.001 |
|  | Model 4 |  | 1.00 | 0.82 (0.76-0.88) | 0.73 (0.67-0.79) | 0.67 (0.61-0.74) | <0.001 |  |  | 0.79 (0.73-0.85) | 0.66 (0.60-0.72) | 0.65 (0.57-0.73) | <0.001 |
| **Ischemic heart disease** | | |  |  |  |  |  |  |  |  |  |  |  |
|  | No. of Deaths | | 359 | 146 | 130 | 104 |  |  | 335 | 223 | 92 | 45 |  |
|  | Model 1 |  | 1.00 | 0.78 (0.64-0.95) | 0.74 (0.59-0.93) | 0.77 (0.59-0.99) | 0.001 |  | 1.00 | 0.71 (0.59-0.84) | 0.60 (0.46-0.77) | 0.47 (0.33-0.67) | <0.001 |
|  | Model 2 |  | 1.00 | 0.79 (0.65-0.96) | 0.76 (0.60-0.96) | 0.79 (0.61-1.02) | 0.004 |  | 1.00 | 0.76 (0.64-0.91) | 0.65 (0.51-0.84) | 0.54 (0.38-0.78) | <0.001 |
|  | Model 3 |  | 1.00 | 0.79 (0.65-0.97) | 0.76 (0.60-0.95) | 0.78 (0.61-1.01) | 0.003 |  | 1.00 | 0.76 (0.64-0.90) | 0.65 (0.51-0.84) | 0.54 (0.38-0.78) | <0.001 |
|  | Model 4 |  | 1.00 | 0.78 (0.64-0.96) | 0.74 (0.59-0.93) | 0.76 (0.59-0.98) | 0.001 |  | 1.00 | 0.75 (0.63-0.90) | 0.64 (0.49-0.82) | 0.53 (0.37-0.76) | <0.001 |
| **Cerebrovascular disease** | | |  |  |  |  |  |  |  |  |  |  |  |
|  | No. of Deaths | | 631 | 255 | 322 | 228 |  |  | 521 | 401 | 223 | 177 |  |
|  | Model 1 |  | 1.00 | 0.70 (0.61-0.82) | 0.69 (0.59-0.80) | 0.62 (0.52-0.73) | <0.001 |  | 1.00 | 0.77 (0.67-0.88) | 0.57 (0.48-0.68) | 0.63 (0.52-0.77) | <0.001 |
|  | Model 2 |  | 1.00 | 0.70 (0.60-0.81) | 0.66 (0.57-0.77) | 0.60 (0.51-0.72) | <0.001 |  | 1.00 | 0.80 (0.70-0.92) | 0.61 (0.51-0.73) | 0.69 (0.56-0.84) | <0.001 |
|  | Model 3 |  | 1.00 | 0.71 (0.61-0.82) | 0.66 (0.56-0.76) | 0.60 (0.50-0.71) | <0.001 |  | 1.00 | 0.81 (0.71-0.92) | 0.62 (0.52-0.74) | 0.69 (0.56-0.85) | <0.001 |
|  | Model 4 |  | 1.00 | 0.71 (0.61-0.82) | 0.66 (0.56-0.77) | 0.60 (0.50-0.72) | <0.001 |  | 1.00 | 0.81 (0.70-0.92) | 0.62 (0.52-0.74) | 0.69 (0.56-0.85) | <0.001 |

*Level of total physical activity was divided into four groups by quartiles, with Q1 as the lowest quartile group. Multivariate models were adjusted for: model 1: age (years); model 2: additionally included level of education (no formal school, primary school, middle school, high school, college, or university or higher); marital status (married, widowed, divorced or separated, or never married); alcohol consumption (non-drinker, occasional drinker, former drinker, or regular drinker); smoking status (never smoker, occasional smoker, former smoker, or regular smoker); intake frequencies of red meat, fruits, and vegetables (daily, 4 to 6 days/wk, 1 to 3 days/wk, monthly, or rarely or never); body-mass index; prevalent diabetes at baseline (presence or absence); family history of heart attack or stroke (presence or absence, only adjusted for in corresponding analysis of cause specific mortality); and menopausal status for women only; model 3: additionally included systolic blood pressure (mmHg); model 4: additionally included leisure sedentary time (h/d).

†Tests for linear trend across physical activity categories were performed by using the continuous physical activity variable in a separate regression model.

# Supplemental Table S5. Associations between domain-specific physical activity and all-cause and cardiovascular mortality*

| Causes of death | | | **Work-related PA** | |  | **Commuting-related PA** | |  | **House-related PA** | |  | **Leisure-time PA** | |
| --- | --- | --- | --- | --- | --- | --- | --- | --- | --- | --- | --- | --- | --- |
|  |  |  | Low | High |  | Low | High |  | Low | High |  | Low | High |
| **All participants** | | |  |  |  |  |  |  |  |  |  |  |  |
|  | No. of Subjects | | 47 784 | 47 200 |  | 49 084 | 45 900 |  | 87 669 | 62 722 |  | 115 061 | 35 330 |
|  | Person years | | 345 222 | 339 126 |  | 354 714 | 329 633 |  | 622 194 | 447 669 |  | 822 631 | 247 232 |
|  | **All causes** | |  |  |  |  |  |  |  |  |  |  |  |
|  |  | No. of Deaths | 3005 | 1894 |  | 2226 | 2673 |  | 6065 | 3641 |  | 7378 | 2328 |
|  |  | Multivariable adjusted HR | 1.00 | 0.85 (0.80-0.91) | | 1.00 | 1.08 (1.02-1.15) | | 1.00 | 0.89 (0.84-0.93) | | 1.00 | 0.86 (0.81-0.91) |
|  | **Ischemic Heart Disease** | |  |  |  |  |  |  |  |  |  |  |  |
|  |  | No. of Deaths | 363 | 202 |  | 253 | 312 |  | 882 | 552 |  | 981 | 453 |
|  |  | Multivariable adjusted HR | 1.00 | 0.99 (0.83-1.20) | | 1.00 | 1.09 (0.90-1.31) | | 1.00 | 0.85 (0.74-0.97) | | 1.00 | 0.94 (0.82-1.08) |
|  | **Cerebrovascular disease** | |  |  |  |  |  |  |  |  |  |  |  |
|  |  | No. of Deaths | 949 | 508 |  | 631 | 826 |  | 1652 | 1106 |  | 2255 | 503 |
|  |  | Multivariable adjusted HR | 1.00 | 0.82 (0.73-0.93) | | 1.00 | 1.01 (0.90-1.13) | | 1.00 | 0.82 (0.75-0.90) | | 1.00 | 0.78 (0.69-0.87) |
| **Men** | | |  |  |  |  |  |  |  |  |  |  |  |
|  | No. of Subjects | | 21 407 | 26 852 |  | 24 856 | 23 403 |  | 57 409 | 6878 |  | 48 154 | 16 133 |
|  | Person years | | 151 456 | 191 441 |  | 176 897 | 166 001 |  | 403 748 | 47 451 |  | 339 103 | 112 095 |
|  | **All causes** | |  |  |  |  |  |  |  |  |  |  |  |
|  |  | No. of Deaths | 1894 | 1358 |  | 1446 | 1806 |  | 4558 | 764 |  | 3967 | 1355 |
|  |  | Multivariable adjusted HR | 1.00 | 0.83 (0.77-0.90) | | 1.00 | 1.10 (1.02-1.19) | | 1.00 | 0.92 (0.85-1.00) | | 1.00 | 0.84 (0.77-0.90) |
|  | **Ischemic Heart Disease** | |  |  |  |  |  |  |  |  |  |  |  |
|  |  | No. of Deaths | 228 | 154 |  | 167 | 215 |  | 644 | 95 |  | 484 | 255 |
|  |  | Multivariable adjusted HR | 1.00 | 1.02 (0.81-1.27) | | 1.00 | 1.08 (0.86-1.35) | | 1.00 | 0.81 (0.65-1.02) | | 1.00 | 0.93 (0.76-1.13) |
|  | **Cerebrovascular disease** | |  |  |  |  |  |  |  |  |  |  |  |
|  |  | No. of Deaths | 587 | 339 |  | 383 | 543 |  | 1235 | 201 |  | 1164 | 272 |
|  |  | Multivariable adjusted HR | 1.00 | 0.77 (0.66-0.89) | | 1.00 | 1.09 (0.94-1.26) | | 1.00 | 0.78 (0.67-0.91) | | 1.00 | 0.70 (0.60-0.83) |
| **Women** | | |  |  |  |  |  |  |  |  |  |  |  |
|  | No. of Subjects | | 26 377 | 20 348 |  | 24 228 | 22 497 |  | 30 260 | 55 844 |  | 66 907 | 19 197 |
|  | Person years | | 193 765 | 147 685 |  | 177 818 | 163 633 |  | 218 446 | 400 219 |  | 483 528 | 135 137 |
|  | **All causes** | |  |  |  |  |  |  |  |  |  |  |  |
|  |  | No. of Deaths | 1111 | 536 |  | 780 | 867 |  | 1507 | 2877 |  | 3411 | 973 |
|  |  | Multivariable adjusted HR | 1.00 | 0.92 (0.82-1.03) | | 1.00 | 1.07 (0.96-1.20) | | 1.00 | 0.85 (0.80-0.91) | | 1.00 | 0.89 (0.82-0.98) |
|  |  |  |  |  | |  |  | |  |  | |  |  |
|  | **Ischemic Heart Disease** | |  |  |  |  |  |  |  |  |  |  |  |
|  |  | No. of Deaths | 135 | 48 |  | 86 | 97 |  | 238 | 457 |  | 497 | 198 |
|  |  | Multivariable adjusted HR | 1.00 | 0.98 (0.68-1.40) | | 1.00 | 1.19 (0.84-1.67) | | 1.00 | 0.84 (0.71-0.99) | | 1.00 | 0.96 (0.78-1.18) |
|  | **Cerebrovascular disease** | |  |  |  |  |  |  |  |  |  |  |  |
|  |  | No. of Deaths | 362 | 169 |  | 248 | 283 |  | 417 | 905 |  | 1091 | 231 |
|  |  | Multivariable adjusted HR | 1.00 | 1.00 (0.82-1.22) | | 1.00 | 0.89 (0.73-1.09) | | 1.00 | 0.85 (0.75-0.96) | | 1.00 | 0.87 (0.73-1.03) |

PA, physical activity.

*Domain-specific physical activity was dichotomized by a median split, with low group used as reference. Multivariate models were adjusted for: age (years); sex (men or women) for all participants only; level of education (no formal school, primary school, middle school, high school, college, or university or higher); marital status (married, widowed, divorced or separated, or never married); alcohol consumption (non-drinker, occasional drinker, former drinker, or regular drinker); smoking status (never smoker, occasional smoker, former smoker, or regular smoker); intake frequencies of red meat, fruits, and vegetables (daily, 4 to 6 days/wk, 1 to 3 days/wk, monthly, or rarely or never); body-mass index; prevalent diabetes at baseline (presence or absence); family history of heart attack or stroke (presence or absence, only adjusted for in corresponding analysis of cause specific mortality); and menopausal status for women only; systolic blood pressure (mmHg); leisure sedentary time (h/d); and all the other domain-specific physical activity.

# Supplemental Table S6. Associations between intensity-specific physical activity and all-cause and cardiovascular mortality*

| Causes of death | | | **Low intensity PA** | |  | **Moderate intensity PA** | |  | **Vigorous intensity PA** | |
| --- | --- | --- | --- | --- | --- | --- | --- | --- | --- | --- |
|  |  |  | Low | High |  | Low | High |  | Low | High |
| **All participants** | | |  |  |  |  |  |  |  |  |
|  | No. of Subjects | | 89 899 | 60 492 |  | 78 668 | 71 723 |  | 112 584 | 37 807 |
|  | Person years | | 635 071 | 434 793 |  | 559 062 | 510 802 |  | 796 270 | 273 593 |
|  | **All causes** | |  |  |  |  |  |  |  |  |
|  |  | No. of Deaths | 6586 | 3120 |  | 5878 | 3828 |  | 7592 | 2114 |
|  |  | Multivariable adjusted HR | 1.00 | 0.84 (0.80-0.88) |  | 1.00 | 0.83 (0.79-0.87) |  | 1.00 | 0.84 (0.79-0.89) |
|  | **Ischemic Heart Disease** | |  |  |  |  |  |  |  |  |
|  |  | No. of Deaths | 1074 | 360 |  | 843 | 591 |  | 1238 | 196 |
|  |  | Multivariable adjusted HR | 1.00 | 0.72 (0.63-0.82) |  | 1.00 | 0.85 (0.75-0.95) |  | 1.00 | 0.83 (0.69-0.99) |
|  | **Cerebrovascular disease** | |  |  |  |  |  |  |  |  |
|  |  | No. of Deaths | 1804 | 954 |  | 1688 | 1070 |  | 2136 | 622 |
|  |  | Multivariable adjusted HR | 1.00 | 0.82 (0.75-0.89) |  | 1.00 | 0.86 (0.79-0.94) |  | 1.00 | 0.83 (0.75-0.93) |
| **Men** | | |  |  |  |  |  |  |  |  |
|  | No. of Subjects | | 45 658 | 18 629 |  | 27 394 | 36 893 |  | 47 020 | 17 267 |
|  | Person years | | 319 390 | 131 808 |  | 190 884 | 260 315 |  | 328 226 | 122 972 |
|  | **All causes** | |  |  |  |  |  |  |  |  |
|  |  | No. of Deaths | 4029 | 1293 |  | 2798 | 2524 |  | 4014 | 1308 |
|  |  | Multivariable adjusted HR | 1.00 | 0.82 (0.77-0.88) |  | 1.00 | 0.82 (0.77-0.87) |  | 1.00 | 0.85 (0.79-0.91) |
|  | **Ischemic Heart Disease** | |  |  |  |  |  |  |  |  |
|  |  | No. of Deaths | 609 | 130 |  | 355 | 384 |  | 613 | 126 |
|  |  | Multivariable adjusted HR | 1.00 | 0.62 (0.51-0.76) |  | 1.00 | 0.88 (0.76-1.03) |  | 1.00 | 0.90 (0.72-1.14) |
|  | **Cerebrovascular disease** | |  |  |  |  |  |  |  |  |
|  |  | No. of Deaths | 1077 | 359 |  | 738 | 698 |  | 1078 | 358 |
|  |  | Multivariable adjusted HR | 1.00 | 0.78 (0.69-0.89) |  | 1.00 | 0.87 (0.78-0.97) |  | 1.00 | 0.81 (0.71-0.94) |
| **Women** | | |  |  |  |  |  |  |  |  |
|  | No. of Subjects | | 44 241 | 41 863 |  | 51 274 | 34 830 |  | 65 564 | 20 540 |
|  | Person years | | 315 680 | 302 985 |  | 368 178 | 250 487 |  | 468 044 | 150 621 |
|  | **Total Mortality** | |  |  |  |  |  |  |  |  |
|  |  | No. of Deaths | 2557 | 1827 |  | 3080 | 1304 |  | 3578 | 806 |
|  |  | Multivariable adjusted HR | 1.00 | 0.84 (0.79-0.90) |  | 1.00 | 0.85 (0.79-0.91) |  | 1.00 | 0.85 (0.77-0.93) |
|  | **Ischemic Heart Disease** | |  |  |  |  |  |  |  |  |
|  |  | No. of Deaths | 465 | 230 |  | 488 | 207 |  | 625 | 70 |
|  |  | Multivariable adjusted HR | 1.00 | 0.78 (0.66-0.94) |  | 1.00 | 0.83 (0.69-0.99) |  | 1.00 | 0.74 (0.54-1.02) |
|  | **Cerebrovascular disease** | |  |  |  |  |  |  |  |  |
|  |  | No. of Deaths | 727 | 595 |  | 950 | 372 |  | 1058 | 264 |
|  |  | Multivariable adjusted HR | 1.00 | 0.83 (0.74-0.94) |  | 1.00 | 0.81 (0.71-0.93) |  | 1.00 | 0.87 (0.73-1.03) |

PA, physical activity.

*Intensity-specific physical activity was dichotomized by a median split, with low group used as reference. Multivariate models were adjusted for: age (years); sex (men or women) for all participants only; level of education (no formal school, primary school, middle school, high school, college, or university or higher); marital status (married, widowed, divorced or separated, or never married); alcohol consumption (non-drinker, occasional drinker, former drinker, or regular drinker); smoking status (never smoker, occasional smoker, former smoker, or regular smoker); intake frequencies of red meat, fruits, and vegetables (daily, 4 to 6 days/wk, 1 to 3 days/wk, monthly, or rarely or never); body-mass index; prevalent diabetes at baseline (presence or absence); family history of heart attack or stroke (presence or absence, only adjusted for in corresponding analysis of cause specific mortality); and menopausal status for women only; systolic blood pressure (mmHg); leisure sedentary time (h/d); and all the other intensity-specific physical activity.

# Supplemental Table S7. Subgroup analysis of associations between total physical activity and total mortality*

| Subgroups | |  | Men | |  | Women | |  | Total | |
| --- | --- | --- | --- | --- | --- | --- | --- | --- | --- | --- |
|  |  |  | HR (95%CI) | ***P_Interaction_*** |  | HR (95%CI) | ***P_Interaction_*** |  | HR (95%CI) | ***P_Interaction_*** |
| All |  |  | 0.75 (0.71-0.81) |  |  | 0.73 (0.68-0.79) |  |  | 0.75 (0.72-0.79) |  |
| Age at baseline | |  |  | 0.992 |  |  | 0.479 |  |  | 0.593 |
|  | <50 |  | 0.72 (0.60-0.87) |  |  | 0.70 (0.55-0.89) |  |  | 0.72 (0.63-0.84) |  |
|  | 50-59 |  | 0.77 (0.68-0.88) |  |  | 0.77 (0.67-0.89) |  |  | 0.77 (0.70-0.85) |  |
|  | ≥60 |  | 0.75 (0.69-0.82) |  |  | 0.72 (0.65-0.80) |  |  | 0.73 (0.69-0.78) |  |
| Region |  |  |  | 0.374 |  |  | 0.471 |  |  | 0.095 |
|  | Rural |  | 0.75 (0.70-0.81) |  |  | 0.74 (0.68-0.81) |  |  | 0.76 (0.71-0.80) |  |
|  | Urban |  | 0.80 (0.71-0.91) |  |  | 0.77 (0.65-0.90) |  |  | 0.80 (0.73-0.89) |  |
| Smoking status | |  |  | 0.349 |  |  | 0.531 |  |  | 0.113 |
|  | Non-current regular smoker |  | 0.78 (0.70-0.87) |  |  | 0.74 (0.69-0.81) |  |  | 0.76 (0.71-0.81) |  |
|  | Current regular smoker |  | 0.76 (0.70-0.83) |  |  | 0.74 (0.51-1.07) |  |  | 0.76 (0.70-0.82) |  |
| Alcohol status | |  |  | 0.425 |  |  | 0.174 |  |  | 0.398 |
|  | Non-current regular drinker |  | 0.79 (0.72-0.85) |  |  | 0.75 (0.69-0.81) |  |  | 0.76 (0.72-0.81) |  |
|  | Current regular drinker |  | 0.69 (0.61-0.77) |  |  | 0.46 (0.26-0.80) |  |  | 0.68 (0.61-0.76) |  |
| Diabetes |  |  |  | 0.326 |  |  | 0.131 |  |  | 0.523 |
|  | No |  | 0.76 (0.71-0.82) |  |  | 0.79 (0.72-0.86) |  |  | 0.77 (0.73-0.81) |  |
|  | Yes |  | 0.79 (0.64-0.97) |  |  | 0.59 (0.49-0.72) |  |  | 0.67 (0.58-0.77) |  |
| BMI |  |  |  | 0.103 |  |  | 0.586 |  |  | 0.334 |
|  | <24.0 |  | 0.74 (0.68-0.81) |  |  | 0.73 (0.65-0.81) |  |  | 0.73 (0.69-0.79) |  |
|  | 24.0-27.9 |  | 0.76 (0.67-0.86) |  |  | 0.77 (0.67-0.89) |  |  | 0.77 (0.70-0.84) |  |
|  | ≥28.0 |  | 0.89 (0.72-1.10) |  |  | 0.75 (0.61-0.92) |  |  | 0.82 (0.71-0.95) |  |
| SBP |  |  |  | 0.388 |  |  | 0.511 |  |  | 0.255 |
|  | <140 |  | 0.79 (0.65-0.96) |  |  | 0.77 (0.61-0.97) |  |  | 0.76 (0.66-0.88) |  |
|  | 140-159 |  | 0.79 (0.71-0.87) |  |  | 0.75 (0.67-0.84) |  |  | 0.77 (0.72-0.83) |  |
|  | ≥160 |  | 0.74 (0.67-0.83) |  |  | 0.74 (0.66-0.84) |  |  | 0.73 (0.68-0.79) |  |
| Work status | |  |  | 0.220 |  |  | 0.002 |  |  | 0.003 |
|  | No |  | 0.72 (0.55-0.96) |  |  | 0.67 (0.56-0.80) |  |  | 0.68 (0.58-0.79) |  |
|  | Yes |  | 0.87 (0.80-0.94) |  |  | 0.95 (0.84-1.08) |  |  | 0.89 (0.83-0.96) |  |
| Vigorous intensity PA | |  |  | 0.384 |  |  | <0.001 |  |  | 0.161 |
|  | =0 MET-h /d |  | 0.79 (0.73-0.85) |  |  | 0.71 (0.65-0.79) |  |  | 0.76 (0.71-0.81) |  |
|  | >0 MET-h /d |  | 0.75 (0.65-0.87) |  |  | 1.37 (1.04-1.82) |  |  | 0.87 (0.76-0.98) |  |
| Leisure sedentary time | |  |  | 0.360 |  |  | 0.772 |  |  | 0.820 |
|  | <3 h/d |  | 0.75 (0.67-0.83) |  |  | 0.76 (0.68-0.86) |  |  | 0.76 (0.70-0.82) |  |
|  | ≥3 h/d |  | 0.77 (0.71-0.84) |  |  | 0.73 (0.66-0.81) |  |  | 0.75 (0.71-0.81) |  |

HR denotes hazard ratio; CI, confidence interval.

*Total physical activity was dichotomized by a median split (15.6 MET-h /d), with low group used as reference. Multivariate models were adjusted for: age (years); sex (men or women) for all participants only; level of education (no formal school, primary school, middle school, high school, college, or university or higher); marital status (married, widowed, divorced or separated, or never married); alcohol consumption (non-drinker, occasional drinker, former drinker, or regular drinker); smoking status (never smoker, occasional smoker, former smoker, or regular smoker); intake frequencies of red meat, fruits, and vegetables (daily, 4 to 6 days/wk, 1 to 3 days/wk, monthly, or rarely or never); body-mass index; prevalent diabetes at baseline (presence or absence); family history of heart attack or stroke (presence or absence, only adjusted for in corresponding analysis of cause specific mortality); and menopausal status for women only; systolic blood pressure (mmHg); and leisure sedentary time (h/d). P values for interactions were computed with the use of likelihood-ratio tests comparing Cox proportional-hazards models with and without cross-product terms for each level of baseline stratifying variable.
